# Spatiotemporal Heterogeneity and Intragenus Variability in Rhizobacterial Associations with *Brassica rapa* Growth

**DOI:** 10.1128/msystems.00060-22

**Published:** 2022-05-16

**Authors:** Scott A. Klasek, Marcus T. Brock, W. John Calder, Hilary G. Morrison, Cynthia Weinig, Lois Maïgnien

**Affiliations:** a Josephine Bay Paul Center, Marine Biological Laboratory, Woods Hole, Massachusetts, USA; b Department of Botany, University of Wyoming, Laramie, Wyoming, USA; c Program in Ecology, University of Wyoming, Laramie, Wyoming, USA; d Department of Molecular Biology, University of Wyoming, Laramie, Wyoming, USA; e Laboratory of Microbiology of Extreme Environments, UMR 6197, Institut Européen de la Mer, Université de Bretagne Occidentale, Plouzane, France; Pacific Northwest National Laboratory

**Keywords:** rhizosphere, microbial community, plant growth promotion, 16S RNA, *Brassica rapa*, feature selection, microbial communities, rhizosphere-inhabiting microbes

## Abstract

Microbial communities in the rhizosphere are distinct from those in soils and are influenced by stochastic and deterministic processes during plant development. These communities contain bacteria capable of promoting growth in host plants through various strategies. While some interactions are characterized in mechanistic detail using model systems, others can be inferred from culture-independent methods, such as 16S amplicon sequencing, using machine learning methods that account for this compositional data type. To characterize assembly processes and identify community members associated with plant growth amid the spatiotemporal variability of the rhizosphere, we grew *Brassica rapa* in a greenhouse time series with amended and reduced microbial treatments. Inoculation with a native soil community increased plant leaf area throughout the time series by up to 28%. Despite identifying spatially and temporally variable amplicon sequence variants (ASVs) in both treatments, inoculated communities were more highly connected and assembled more deterministically overall. Using a generalized linear modeling approach controlling for spatial variability, we identified 43 unique ASVs that were positively or negatively associated with leaf area, biomass, or growth rates across treatments and time stages. ASVs of the genus *Flavobacterium* dominated rhizosphere communities and showed some of the strongest positive and negative correlations with plant growth. Members of this genus, and growth-associated ASVs more broadly, exhibited variable connectivity in networks independent of growth association (positive or negative). These findings suggest host-rhizobacterial interactions vary temporally at narrow taxonomic scales and present a framework for identifying rhizobacteria that may work independently or in concert to improve agricultural yields.

**IMPORTANCE** The rhizosphere, the zone of soil surrounding plant roots, is a hot spot for microbial activity, hosting bacteria capable of promoting plant growth in ways like increasing nutrient availability or fighting plant pathogens. This microbial system is highly diverse and most bacteria are unculturable, so to identify specific bacteria associated with plant growth, we used culture-independent community DNA sequencing combined with machine learning techniques. We identified 43 specific bacterial sequences associated with the growth of the plant *Brassica rapa* in different soil microbial treatments and at different stages of plant development. Most associations between bacterial abundances and plant growth were positive, although similar bacterial groups sometimes had different effects on growth. Why this happens will require more research, but overall, this study provides a way to identify native bacteria from plant roots that might be isolated and applied to boost agricultural yields.

## INTRODUCTION

Millimeters in thickness, the rhizosphere is the zone of soil that surrounds plant roots and supports bacterial communities distinct from those in bulk soils (1, 2). Plants induce this “rhizosphere effect” by exuding organic compounds from roots, which recruit rhizosphere bacteria and change bacterial community structure (3, 4). These rhizobacteria can enhance plant growth directly or indirectly by mobilizing soil nutrients (5, 6), suppressing disease (7) and herbivory (8, 9), mediating hormone signaling (10), and increasing tolerance to physical stresses such as drought (11) and salinity (12). Leveraging these plant growth-promoting rhizobacteria (PGPR) and their microbial communities to increase agricultural output is of great interest and importance (13–15).

Rhizosphere microbial communities are complex and spatiotemporally variable and can interact with hosts and with each other to maintain plant health in ways that individual isolates cannot (16, 17). Although rhizosphere composition varies to some extent across host species (18), host genetic and epigenetic regulatory mechanisms, including the circadian clock and histone methylation, can affect rhizosphere recruitment (19, 20). Variations in root exudate profiles across *Arabidopsis* growth stages have been interpreted to change rhizobacterial community composition (21), while subsequent investigations have detailed how certain exudate compounds can recruit or inhibit specific bacterial taxa within communities, often in response to nutrient stress (22–24). Population-level spatiotemporal variability has also been observed, such as in a reporter assay where the activity of a root-associated Pseudomonas aligned with expected rhizodeposition patterns during root growth (25). Not only guided by host influences, the assembly of plant-associated bacterial communities is to an extent shaped by stochastic forces across small spatial or temporal scales (26–28).

Considering this, high-resolution sampling is required to disentangle specific drivers of rhizosphere community assembly.

Several strategies have been used to identify plant growth-promoting rhizobacterial community sequence data (29, 30). Another used a random forest approach to identify a set of 75 operational taxonomic units (OTUs) whose relative abundances could explain 31% of the difference in grain weight of foxtail millet (31). Although the human microbiome literature has employed a variety of machine-learning strategies for identifying microbial community features (taxa or genes) associated with health or disease classifications (32), fewer studies have used these strategies to identify associations between rhizosphere community members and continuous plant growth data.

To identify and characterize plant growth-promoting bacteria within the context of rhizosphere community dynamics, we grew *Brassica rapa* in a 2-week time series greenhouse experiment using two soil treatments. These consisted of an autoclaved soil matrix that was inoculated with a plant growth-promoting soil (South Brush Creek [SBC], inoculated treatment) or left to allow microbial community recolonization by atmospheric deposition (disrupted treatment). Rhizosphere communities were sampled between 2 and 14 days of growth across six replicates (spatial blocks) and across diel cycles. Using an original approach based on generalized linear and Bayesian multilevel modeling, we identified 16S rRNA amplicon sequence variants (ASVs), ASV linear combinations, or clusters of cooccurring ASVs (network modules) whose abundances best predict leaf area, plant biomass, and growth rates across early or late stages of growth. Furthermore, we characterized cooccurrence patterns of growth-associated ASVs that may reveal their potential for plant growth promotion in field studies.

## RESULTS

### Soil inoculum promotes *Brassica rapa* growth.

**FIG 1:**
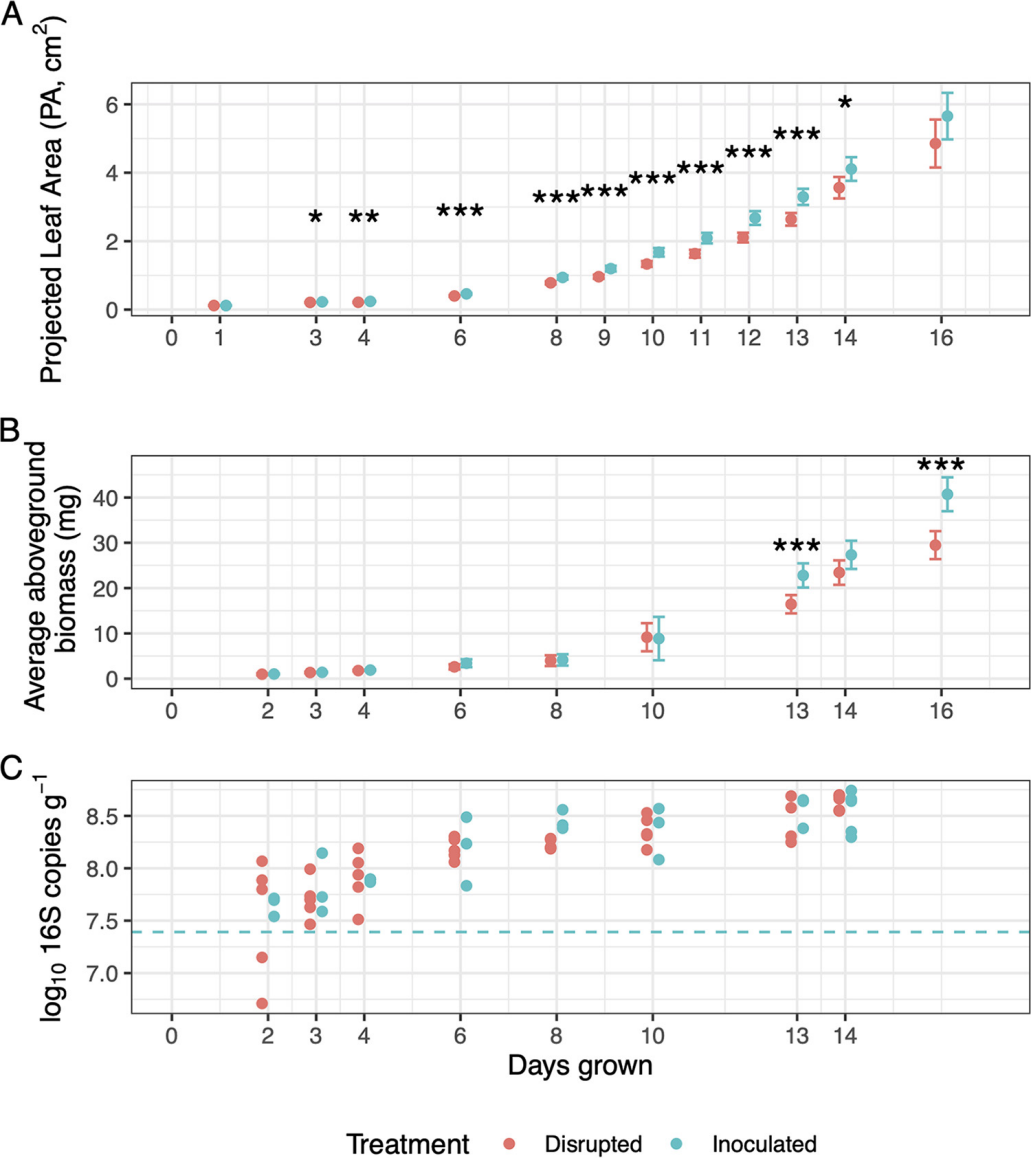
Plant and bacterial growth across the time series. (A and B) Projected leaf area (PA) (A) and aboveground biomass (B) by treatment across the time series. Points represent the mean across six blocks and five different harvest times (on days 3, 4, 13, or 14, with 95% confidence intervals shown). *, *P* < 0.05; **, *P* < 0.01; ***, *P* < 0.001. (C) Bacterial 16S gene counts per gram rhizosphere soil. Dashed line indicates mean SBC soil 16S rRNA gene copy number at inoculation, accounting for dilution.

*B. rapa* was grown in two soil settings to examine plant growth promotion (PGP) under different modes of community assembly, in an autoclaved soil favoring a more random assembly through migration and colonization of air/water-sourced microorganisms (disrupted treatment) or in an autoclaved soil followed by inoculation of a soil slurry favoring a more deterministic assembly (inoculated treatment). Compared to plants grown in disrupted soil treatments, inoculation of the *B. rapa* accession Wisconsin FastPlant (FPsc) with SBC soil resulted in higher average leaf projected area (PA) on day three after germination, persisting until day 14 (Fig. 1A). This effect was highest at day 11, when PA of inoculated plants was 28% higher than that of plants grown in the disrupted soil treatment. By around day 12, leaves began to self-shade, decreasing the correlation between PA and true leaf area. A 39% increase in aboveground biomass was observed by day 13 (Fig. 1B), an effect that was not significant earlier in the time series. This is likely a consequence of fewer measurements taken during destructive sampling. Relative growth rates (RGR), calculated from slopes of log-transformed PA measurements from consecutive intervals, were higher in inoculated compared to disrupted treatments throughout the time series (two-tailed *t* test *P* = 0.0399; also see Fig. S1 at https://doi.org/10.6084/m9.figshare.c.5787851.v1). Measurements of aboveground biomass and PA at harvest correlated highly across all cultivation pots (adjusted *R*^2^ = 0.94). In addition, we observed differences in growth across the six blocks within the greenhouse in each treatment. These block effects were noted with PA measurements from days 3, 10/11, and 13 (two-way analyses of variance [ANOVAs], maximum *P* = 0.0288). Block variation in PA measurements averaged on days 10 and 11 is shown in Fig. S2 at https://doi.org/10.6084/m9.figshare.c.5787851.v1. Block differences in biomass and relative growth rates showed less consistent patterns. Concentrations of bacterial 16S rRNA gene copies increased with time until around day 8 but did not significantly vary across treatments (Fig. 1C). Plants showed no indication of disease.

### Inoculated rhizospheres assemble deterministically.

**FIG 2:**
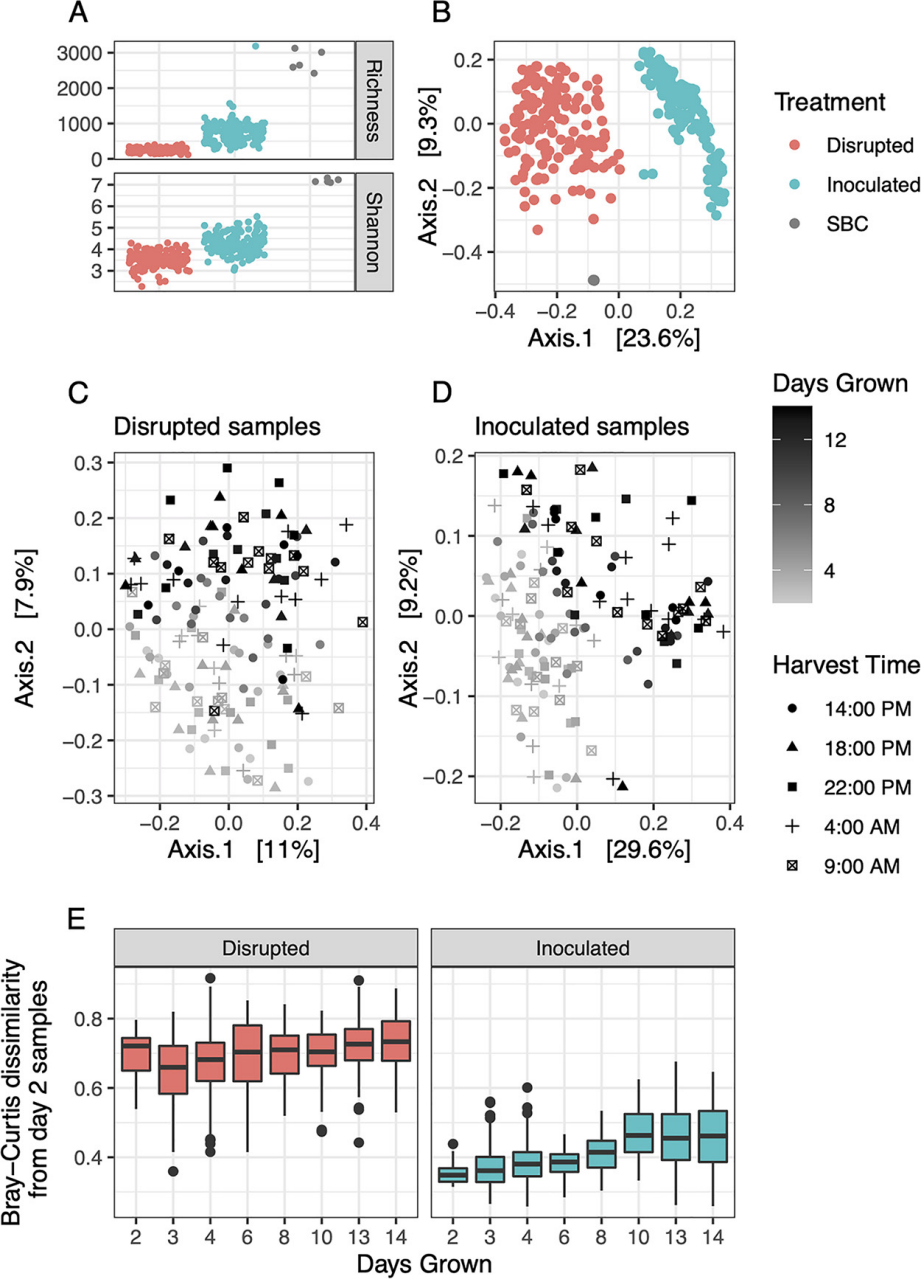
Rhizosphere bacterial community diversity. (A and B) Alpha diversity (number of observed ASVs and Shannon indices) (A) and PCoA ordination of Bray-Curtis dissimilarities (B) between raw soil inoculum (SBC) and rhizosphere communities by treatment. Separate ordinations of disrupted (C) and inoculated (D) communities are shaded by the number of days of plant growth, with harvest times indicated by shape. (E) Bray-Curtis dissimilarities across the time series. In each treatment, dissimilarities from communities harvested at each day are shown relative to those from day 2. (Dissimilarities at day 2 were calculated from day 2 samples only.) Raw read counts were transformed using cumulative sum scaling.

Inoculated rhizosphere communities showed higher richness and evenness compared to disrupted ones, as measured by numbers of observed ASVs and Shannon’s diversity indices (Fig. 2A). SBC inoculum communities were even more diverse. The top 100 ASVs from SBC soil inoculum communities included 15 bacterial classes in near-equal proportions, consisting of around 30% of sequence reads (Fig. S3A at https://doi.org/10.6084/m9.figshare.c.5787851.v1). In contrast, 2 days after germination, the 100 most abundant ASVs in rhizosphere communities of both treatments consisted of only four classes (*Actinobacteria*, *Bacilli*, *Bacteroidia*, and *Gammaproteobacteria*) and contained roughly 80% of sequences (Fig. S3B at https://doi.org/10.6084/m9.figshare.c.5787851.v1). Neither metric of alpha diversity at the community level varied notably across time. Treatment accounted for 22% of the variation in rhizosphere community structure (permutational multivariate ANOVA [PERMANOVA], *P* < 0.001), an observation reflected by principal coordinate analysis (PCoA) ordinations of Bray-Curtis dissimilarities (Fig. 2B). Days of growth across the 14-day time series explained 5.8% and 16.8% of community variation in disrupted and inoculated communities respectively (PERMANOVA, *P* < 0.001 for both), and separation between early and late stages of growth was more evident in inoculated communities (Fig. 2C and D). In rhizospheres intensively sampled at five times per day during two 48-h cycles (3 to 4 and 13 to 14 days), harvest times did not influence community structure across either treatment (Fig. 2C and D).

Several measures point toward deterministic community assembly in rhizospheres of inoculated treatments. Rhizosphere bacterial communities from disrupted treatments exhibited high but consistent dissimilarity throughout the time series, while inoculated communities showed low but increasing dissimilarity (relative to day 2 samples) until day 10 (Fig. 2E). Abundant ASVs were more highly prevalent (present in a higher percentage of communities) across inoculated communities than disrupted ones (Fig. S4 at https://doi.org/10.6084/m9.figshare.c.5787851.v1). The core community of the inoculated treatment consisted of 46 ASVs present across all 144 samples and accounting for 51% of all sequence reads, while only 8 ASVs (36% of reads) were present in all 144 disrupted communities. Cooccurrence networks were constructed from rhizosphere communities belonging to the same treatment and time stage (here referred to as sample groups). Early groups correspond to rhizospheres at 3 to 4 days of growth and late groups to 13 to 14 days of growth, chosen to represent the increased sampling frequency within these 48-h intervals. Networks from all inoculated rhizospheres contained more connections, and vertices (ASVs) were more highly interconnected than in all the disrupted ones (Table 1). The mean number (degree) and strength of connections also increased conspicuously between early and late networks of inoculated communities only. Numbers of modules, or clusters of cooccurring ASVs in each sample group, ranged from 54 to 222 (Table 1).

**TABLE 1:** Co-occurrence network summary statistics for six sample groups^*a*^

Sample group	Count	No. of vertices	Mean degree	Mean strength	No. of edges	Mean wt	No. of modules
All inoculated	144	718	19.98	0.798	7,173	0.040	56
All disrupted	144	548	9.26	0.620	2,537	0.067	54
Early inoculated	60	614	4.78	0.108	1,468	0.022	222
Late inoculated	60	787	13.88	0.652	5,463	0.047	70
Early disrupted	60	423	5.35	0.405	1,132	0.076	83
Late disrupted	60	469	6.49	0.315	1,522	0.048	59

a Counts display the number of communities used for constructing networks, and vertices are the number of ASVs in each network. Degree and strength are attributes of vertices that represent the number of connections and sum of edge weights from each vertex. Edges are the number of co-occurrences in each graph, and weights are attributes of edges that represent cooccurrence strength. Modules of co-occurring ASVs are shown for each graph after removing negative edges.

Rhizosphere communities from both treatments exhibited spatial variation across the six greenhouse blocks, with block membership explaining 14% and 17% of variance among inoculated and disrupted communities, respectively (PERMANOVA, *P* < 0.001 for both). Differential abundance analysis (DESeq2 [33]) identified 33 ASVs whose abundances varied significantly across 4 of 6 blocks in inoculated communities (Fig. S5 at https://doi.org/10.6084/m9.figshare.c.5787851.v1) and 49 ASVs across 5 of 6 blocks in disrupted communities (Fig. S6 at https://doi.org/10.6084/m9.figshare.c.5787851.v1). These block-specific biomarkers made up highly variable proportions of their respective communities and included several dominant ASVs, particularly in the genera *Flavobacterium* and *Paenibacillus*. In both treatments, block 6 had highest numbers and abundances of block-specific biomarkers (Fig. S5 and S6 at https://doi.org/10.6084/m9.figshare.c.5787851.v1). ASVs whose abundances varied by growth stage (early or late, harvested before or after 7 days of growth) made up similar proportions of inoculated and disrupted rhizosphere communities (Fig. S7A at https://doi.org/10.6084/m9.figshare.c.5787851.v1). However, higher total numbers of growth-stage biomarker ASVs in inoculated communities likely reflect the higher alpha diversity (Fig. 2A). ASVs whose abundance peaked at one particular day of the time series made up smaller proportions of both treatments than growth-stage biomarker ASVs (Fig. S7B) but were more abundant in disrupted rhizospheres, recapitulating their higher degree of stochastic assembly. All ASVs that varied in differential abundance across treatment, block, treatment × time (growth stage or specific day), or treatment × block are presented as a table of logical values (Table S1 at https://doi.org/10.6084/m9.figshare.c.5787851.v1). In both treatments, *Flavobacterium* represented the most diverse and the most abundant genus within rhizosphere communities, containing 630 ASVs and over 40% of reads. Overall, reads assigned to *Flavobacterium* increased in abundance with time in disrupted communities but decreased in inoculated ones (Fig. S8 at https://doi.org/10.6084/m9.figshare.c.5787851.v1).

### Rhizobacterial features associated with plant growth.

**FIG 3:**
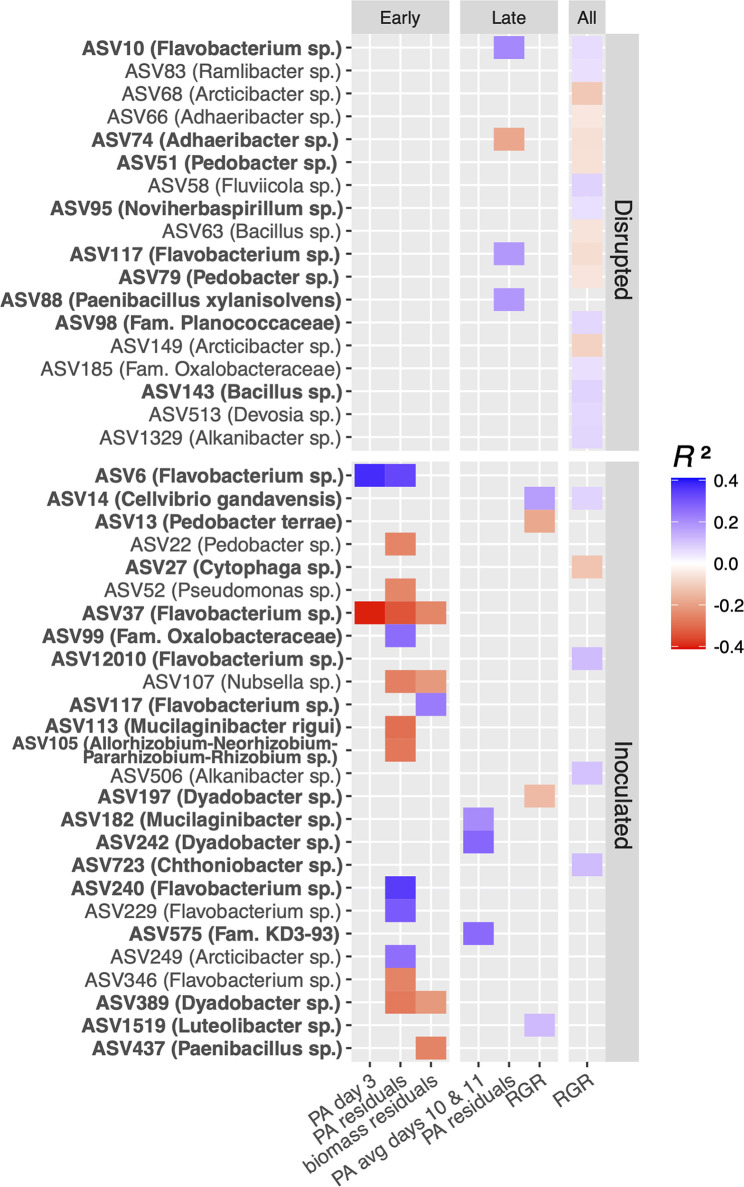
ASVs associated with plant growth in both treatments. ASVs were associated with projected area (PA), residuals of aboveground biomass controlling for days grown, residuals of PA controlling for days grown and sample collection date, and relative growth rates (RGR). Measurements from early and late columns denote rhizosphere communities from plants grown 3 to 4 or 13 to 14 days, respectively, while All denotes all plants grown >2 days. *R*^2^ values show proportions of variance explained by Bayesian models of abundances of each ASV versus plant growth, with blue showing positive associations and red being negative. ASVs are labeled by most specific taxonomy information and shown in bold if also included in best multivariate Bayesian models. PA, residual, and RGR values represent averages from 3 to 7 plants (early stage) or 2 plants (late stage).

We used generalized linear modeling (GLM) to identify associations between normalized abundances of ASV or modules (here features) and numerical plant growth measurements while controlling for block heterogeneity. A least absolute selection and shrinkage operator (LASSO) was applied to refine the number of important, nonredundant predictive features. Rhizosphere communities were classified into sample groups based on inoculation treatment and time (early, 3 or 4 days growth; late, 13 or 14 days growth). GLM runs consisted of ASV or module abundances from these communities paired with contemporaneous plant growth data (Table 2). Temporal variation in RGR values between days 3 and 4 (Fig. S1 at https://doi.org/10.6084/m9.figshare.c.5787851.v1) prevented us from associating RGR with features in early-stage communities. After feature selection, Bayesian multilevel modeling (of individual features or linear combinations thereof) reduced the number of growth-associated ASVs or modules to only those that were most unambiguously associated with plant growth (i.e., nonzero slopes of feature abundances versus plant growth; Table S2 at https://doi.org/10.6084/m9.figshare.c.5787851.v1). Further information is provided in Materials and Methods. This statistical framework allowed us to identify a total of 43 unique ASVs from rhizosphere communities that were good predictors of at least one measurement of plant growth across different time stages and treatments (here growth positive or growth negative) (Fig. 3). Twenty-six were associated with inoculated communities, 18 with disrupted communities, and only one (ASV117) with both. Abundances of individual ASVs explained from 5% to 40% of the variance in plant growth data (Table S2), while models containing linear combinations of multiple ASVs explained from 18% to 84% of variance in growth (conditional *R*^2^) (Table S3 at https://doi.org/10.6084/m9.figshare.c.5787851.v1). Seven growth-associated ASVs were associated with more than one growth metric, four of which were assigned to the genus *Flavobacterium* (Fig. 3). In general, ASVs associated with RGR explained less variance than those associated with other growth measurements. More traditional biomarker detection techniques, such as DESeq2 (33), applied to rhizosphere communities classified as growth-promoting, possibly growth-promoting, or non-growth-promoting categories did not return any significant results, justifying our GLM approach (for more detail, see Materials and Methods).

**TABLE 2:** Associations of rhizosphere communities and plant growth data tested in the GLM^*a*^

Time stage	Response
Early (days 3 and 4)	Leaf PA, day 3
Early	PA residuals from days 3 and 4
Early	Biomass residuals from days 3 and 4
Late (days 13 and 14)	Avg PA, days 10 and 11
Late	PA, day 13
Late	PA residuals from days 13 and 14
Late	Biomass residuals from days 13 and 14
Late	RGR
All, from 3 days onwards	RGR

a These associations were run for both inoculated and disrupted soil treatments and with ASV and module abundances. RGR measurements were consistent across the time series (see Fig. S1 at https://doi.org/10.6084/m9.figshare.c.5787851.v1), allowing us to include communities from across the time series (excluding those from day 2, when only one PA measurement was available).

**FIG 4:**
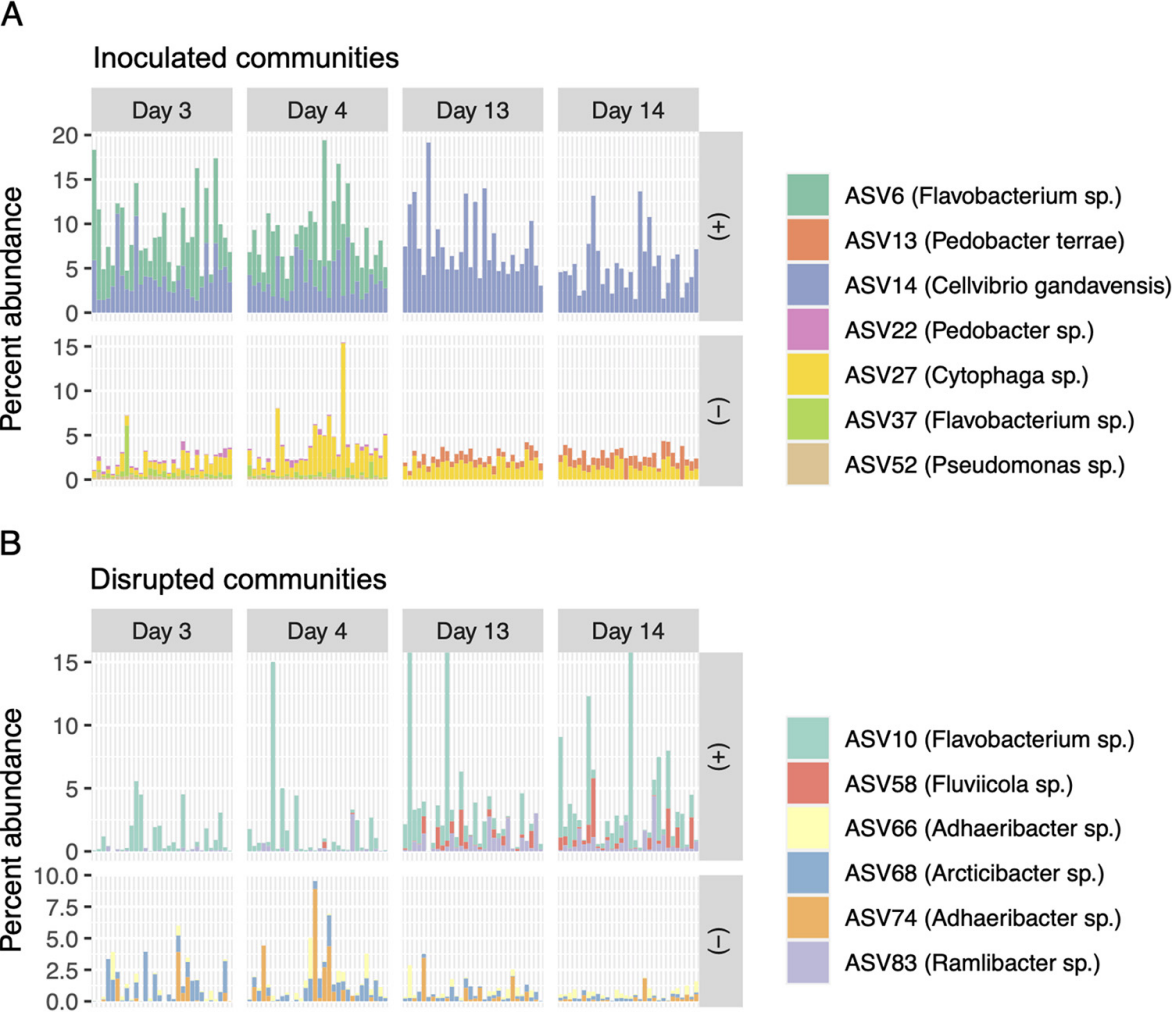
Most abundant growth-associated ASVs across time. (A and B) Relative abundances of growth-associated ASVs from inoculated (A) or disrupted (B) communities harvested at times where they were positively or negatively associated with any metric of plant growth (early, days 3 and 4; late, days 13 and 14). ASVs with abundances of less than 0.2% of communities, on average, by treatment and time were omitted. Most specific taxonomies are shown. The *y* axis for positive growth-associated ASVs in panel B was truncated because ASV10 exceeded 15% abundance within three disrupted communities, with a maximum abundance of 36.6%.

Eight ASVs belonging to the genus *Flavobacterium* were associated with growth across different treatments and time stages, several of which demonstrated particularly strong associations with growth (Fig. 3) and were highly abundant members of rhizosphere communities (Fig. 4). While most of these were growth positive, ASV37 was negatively associated with PA, biomass residuals, and PA residuals in early inoculated communities (Fig. 3). Flavobacterium ASV sequences did not cluster phylogenetically with growth associations in an obvious pattern, although three of the five unambiguously growth-positive ASVs grouped into a distinct subclade based on 16S rRNA V4V5 sequences (Fig. S9 at https://doi.org/10.6084/m9.figshare.c.5787851.v1). Despite the high correlation (*R*^2^ = 0.85) between PA values from inoculated plants at day 3 and residuals of PA from days 3 and 4, 11 additional ASVs were associated with PA residuals. Interestingly, ASV117, a *Flavobacterium* with a greater than 99% sequence identity to ASV37, was the only sequence that was associated both positively and negatively with growth and associated with growth in both inoculated and disrupted treatments. Five other genera (*Arcticibacter*, *Bacillus*, *Dyadobacter*, *Mucilaginibacter*, and *Paenibacillus*) also contained multiple ASVs that showed either positive or negative associations with growth (Fig. 3). These were identified across different treatments, stages, and measurements of plant growth but also sometimes within the same pairings of sample groups and plant growth data (*Flavobacterium* in early-stage inoculated treatments, *Bacillus* with RGR in disrupted treatments).

Overall, relative abundances of ASVs associated with any measurement of growth were higher in inoculated than disrupted communities, and relative abundances of all growth-positive ASVs were higher than those of growth-negative ones in both treatments (Fig. 4). In addition to *Flavobacterium*, these dominant growth-associated ASVs included members of *Cellvibrio*, *Cytophaga*, *Adhaeribacter*, and *Pedobacter*. All dominant growth-associated ASVs in disrupted communities were associated with RGR throughout the entirety of the time series. Relative abundances of growth-positive ASVs showed high variability in disrupted communities, with *Flavobacterium* ASV10 ranging from less than 0.1% to 36% of reads and *Fluviicola* ASV58 from 0 to 4.6% (Fig. 4).

**FIG 5:**
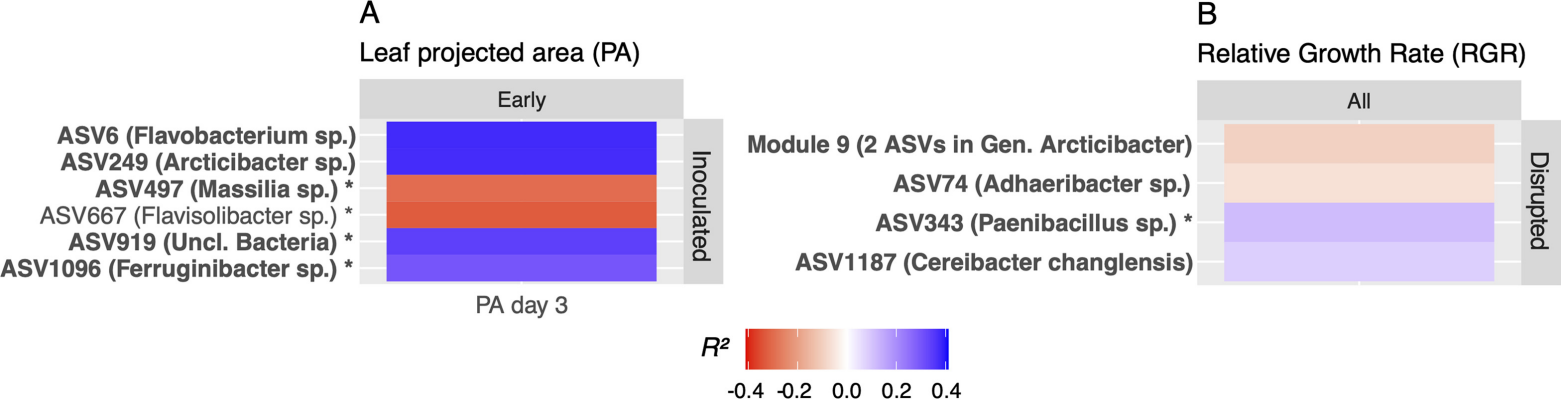
Modules associated with plant growth in both treatments. Modules were associated with PA (A) and RGR (B). Columns denote time stages when rhizosphere communities were sampled (early, 3 to 4 days; all, > 2 days). *R*^2^ values show proportions of variance explained by Bayesian models of each individual module’s abundance versus plant growth, with blue showing positive associations and red being negative. Modules consisting of only one ASV show its most specific taxonomy, and modules indicated with an asterisk denote ASVs that were omitted from the ASV-centered workflow based on low abundance or prevalence. Module labels are shown in boldface if also included in best multivariate Bayesian models. PA, residual, and RGR values represent the averages from 2 to 7 plants.

We then searched for growth-associated modules as described for ASVs. By condensing thousands of ASVs into dozens or hundreds of modules, we were able to include ASVs with low abundances or prevalences that would otherwise have been omitted from the GLM (see Materials and Methods for more detail). We identified 10 module associations with growth, where all modules consisted of only one or two ASVs (Fig. 5). Five of these consisted of low-prevalence or low-abundance ASVs omitted from the ASV-centric approach, while others included highly abundant singleton modules, such as *Flavobacterium* ASV6 and *Adhaeribacter* ASV74. Three of the four modules consisting of higher-abundance singleton ASVs revealed associations to PA or PA residuals similar to those shown in Fig. 3, and the two *Arcticibacter* ASVs in module 9 (ASVs 68 and 149) (Fig. 5B) both showed negative associations with RGR as individuals (Fig. 3).

### Co-occurrence networks.

**FIG 6:**
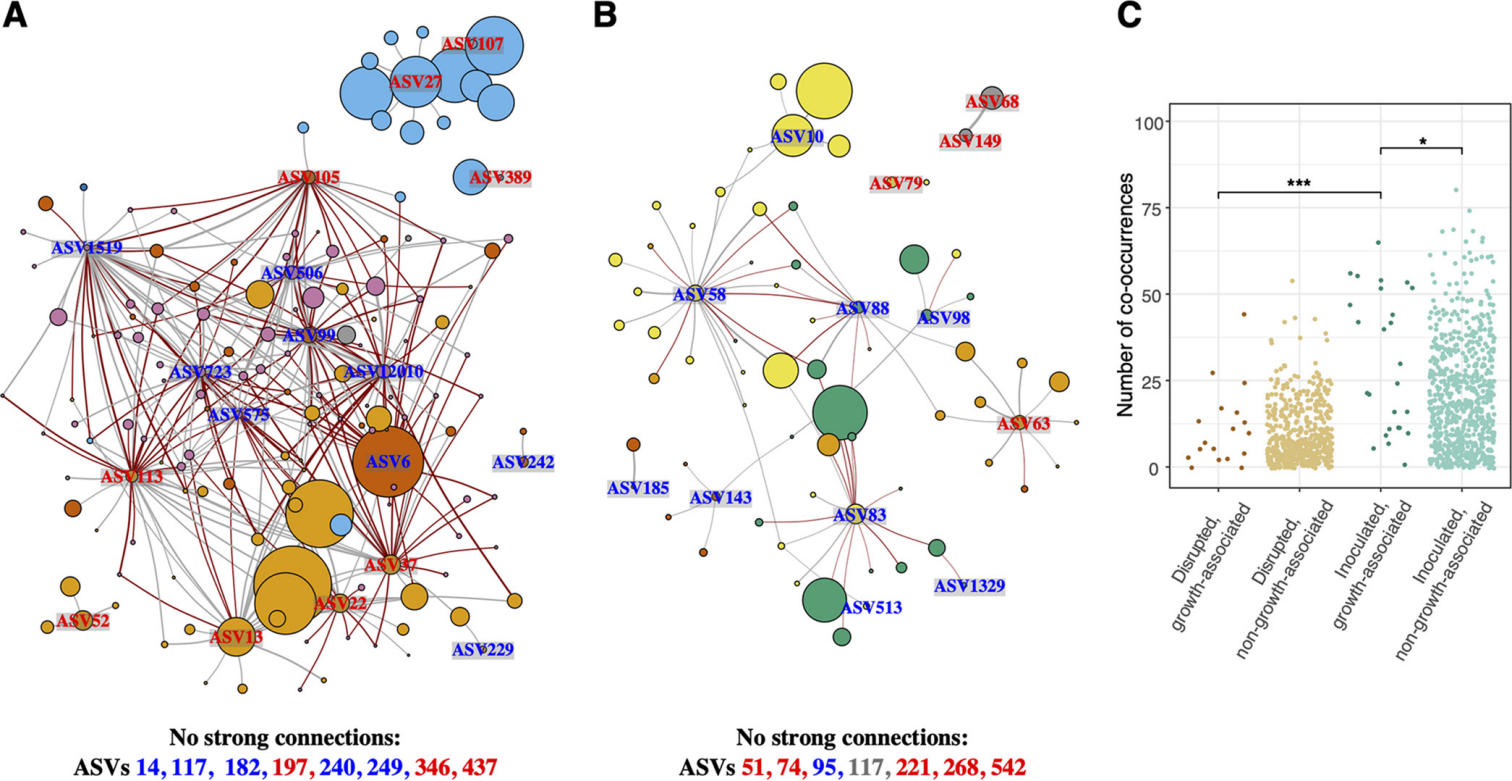
Subsetted networks showing growth-associated ASVs and their strong co-occurrences. Networks show inoculated (A) and disrupted (B) communities, subsetted to include only growth-associated ASVs and those they show strong co-occurrences with. Positive co-occurrences are shown as gray edges for weights exceeding the third quartile of all weights from unsubsetted networks, and negative co-occurrences are shown as red edges for weights below the first quartile. Nodes labeled in blue are ASVs positively associated with growth, and nodes labeled in red are negative. Unlabeled nodes are connected to one or more growth-associated ASVs. Node size corresponds to the square-rooted average relative abundance, and node color indicates module membership. Edge thickness corresponds to connection weight. Node sizes and edge widths across networks are not to scale. (C) Degree (number of connections) from each ASV in unsubsetted networks of inoculated or disrupted communities, separated by whether they were associated with growth. *, *P* < 0.05; ***, *P* < 0.001.

Co-occurrence networks of growth-associated ASVs, showing ASVs displaying strong direct positive or negative connections to them, revealed differences in connectivity across treatments (Fig. 6). The inoculated network (Fig. 6A) consisted of 328 edges connecting 155 ASVs from six modules that made up an average of 39% of the reads within communities, while the disrupted network (Fig. 6B) contained 76 edges connecting 70 ASVs from six modules that made up 19% of reads. Several dominant growth-associated ASVs from both treatments (particularly Cellvibrio gandavensis ASV14 and *Adhaeribacter* ASV74 in inoculated and disrupted communities, respectively) showed no strong connections and were removed from network graphs. Certain modules within both networks contained both growth-positive and growth-negative ASVs, although growth-associated ASVs within these modules were generally weighted toward one direction. Some ASVs in both networks showed positive co-occurrences with both growth-positive and growth-negative ASVs. These comprised 50 ASVs across 26 genera in inoculated communities and two ASVs from the genus *Paenibacillus* in disrupted rhizospheres. Considering all (not just strong) co-occurrences, growth-associated ASVs from inoculated communities were more highly connected than those from disrupted communities and non-growth-associated ASVs from inoculated communities (Fig. 6C). Nevertheless, growth-positive and growth-negative ASVs showed no differences in connectivity in either network, and neither did ASVs that were kept versus omitted in multivariate Bayesian models.

## DISCUSSION

The use of two soil treatments, consisting of a sterilized soil matrix that was either amended with a raw soil inoculum (inoculated) or exposed to colonization by atmospheric deposition (disrupted), allowed us to examine the plant growth-promoting potential of a native soil bacterial community relative to a recent assemblage sourced from greenhouse air and/or water. While 16S rRNA gene copy numbers are not indicative of live bacterial biomass, similar increases in copy numbers over time in both treatments allow us to discount biomass as a confounding factor. Trial experiments demonstrated no discernible difference in PA or aboveground biomass between plants grown in disrupted or freshly autoclaved soils, supporting the role of an intact microbial community in *B. rapa* growth promotion (data not shown). Maximum percent yield increases of 28 to 39% (leaf PA and aboveground biomass, respectively) in inoculated plants were within range of several inocula from agricultural and forest soils that largely increased *Arabidopsis* shoot and root biomass relative to axenic controls (34). Temporal changes in rhizobacterial community composition across stages of plant growth have been described previously (35, 36) and specifically attributed to changes in root exudate chemistry (37) or microbial succession independent of host growth stage (38). Although some exudates in *Arabidopsis* and other members of *Brassicaceae* (notably glucosinolates) exhibit circadian fluctuation, many do not (39, 40). Although glucosinolate exudation patterns in FPsc are uncharacterized, differences in growing conditions (such as between greenhouse and growth chamber experiments) may partially explain the consistency we observed in rhizosphere community composition across diel cycles. The separation in community beta-diversity in both treatments between weeks 1 and 2 (Fig. 2C and D) occurred during thinning, which may be a result of density-dependent changes in root exudation between day 6 and later time points.

We interpret the contrasting patterns of beta-diversity and network connectivity of rhizosphere communities from disrupted and inoculated treatments as consequences of stochastic versus deterministic assembly. Although rhizosphere microbial community assembly is driven by both processes (41–43), the high dissimilarity in disrupted community beta-diversity over time (Fig. 2E) has also been noted in soils after wildfire disturbance (44). The corresponding reduction in plant growth associated with disturbance (sterilization) and recolonization highlights the importance of deterministic processes of rhizosphere community assembly for agricultural contexts.

In addition, our observation of several ASVs with differential abundance patterns across greenhouse blocks in both treatments suggests that random or dispersal forces also shaped rhizosphere community assembly, as previously noted in *Arabidopsis* phyllospheres and across meter-scale field plots in rhizospheres of several *Brassica* cultivars (26, 27). Alternatively, subtle and unmeasured variations in temperature or other factors across different areas of the greenhouse may play a role (particularly for block 6, which sat directly underneath a heater; see Fig. S5 and S6 at https://doi.org/10.6084/m9.figshare.c.5787851.v1). It is important to note that whole-rhizosphere sampling as described here may obscure fine-scale spatial distributions of bacterial populations along the root axis, such as those recently linked with root flavone exudation during nitrogen deprivation (22).

Roles of growth-associated rhizobacteria may be direct (such as aiding nutrient uptake via N, P mineralization, or in competing with plants for these nutrients) or indirect, such as through hormonal suppression of root growth-inhibiting bacteria by *Variovorax* (10). Random forest associations have been used to identify bacterial OTUs, genera, and taxonomic orders positively and negatively associated with grain weight in foxtail millet (31), growth of tomato plants (45), and soybean yields (46). In these studies, the proportion of variance (*R*^2^) attributable to combinations of these identified features ranged from 0.31 to 0.38, which was on the lower end of the variance explained by our multivariate Bayesian models containing multiple features (mean *R*^2^ = 0.4; Table S3 at https://doi.org/10.6084/m9.figshare.c.5787851.v1). Although our decision to select features at the ASV level risks dispersing slightly different 16S rRNA gene sequences that may originate from the same genome into separate entities (47), this approach addresses strain-level heterogeneity that may provide insight into variability in rhizosphere functional potential (48–50). Connectivity patterns allow us to at least partially resolve this issue: despite sharing 99.2% sequence identity, two *Flavobacterium* ASVs (6 and 37) that negatively cooccur with each other and have different effects on plant growth (Fig. 6A) likely represent different strains, while two positively cooccurring *Arcticibacter* ASVs (68 and 149, with 99.3% sequence identity) that belong to the same module (Fig. 6B) likely arise from the same genome.

While our approach primarily focused on abundant members of rhizobacterial communities, rare members of the rhizosphere can contribute to maintenance of plant health, such as in producing compounds to suppress fungal pathogens (51). The higher statistical power inherent to more prevalent features (those detected in higher percentages of communities) likely allowed them to be more easily identified in GLM LASSO runs. Nevertheless, clustering ASVs into modules allowed us to detect several associations between plant growth and lower-abundance rhizosphere members.

Many growth associations we found were specific to early or late stages of plant growth only, as others have observed in PGPR isolates from banana rhizospheres (52). In particular, we identified seven early-stage biomarkers associated with growth within the time stages in which they were most abundant (ASVs 6, 37, 99, 113, 105, and 249). Contemporaneous associations in late stages or disrupted treatments were less clear and identified lower-abundance ASVs overall, suggesting that early stages of plant growth are particularly influenced by members of diverse or stable rhizosphere communities. Despite the considerable spatial heterogeneity across rhizobacterial communities (Fig. S5 and S6), only four growth-associated ASVs varied significantly across greenhouse blocks, of which only ASV13 (genus *Pedobacter*) exceeded 0.2% average abundance. The overall lack of variability across spatial blocks in growth-associated features appears to reflect deterministic assembly processes (even in disrupted communities), which may be driven by selection in response to root exudation patterns.

Imperfect correlations between different growth metrics may account for differences in features with which they were associated. In particular, PA from inoculated plants at day 3 and PA residuals at days 3 and 4 showed an *R*^2^ of 0.85 and were associated with ASVs 6 and 37, yet several additional ASVs were associated with PA residuals only (Fig. 3). This may highlight the sensitivity of the GLM in selecting features from similar or highly correlated data types, making the choice of growth data a critical consideration. Although most growth-associated features detected in univariate Bayesian models were also significant in multivariate models, the ones that were significant only in a multivariate context (Table S2 at https://doi.org/10.6084/m9.figshare.c.5787851.v1) may only appear so when controlling for the abundances of other ASVs or may require each other to synergistically affect growth.

Members of *Flavobacterium* have been associated with increased potato root and shoot growth in one of the first high-throughput sequencing studies of the rhizosphere (29). Plant-associated members of this genus are capable of gliding motility, possess enzymes to degrade glycans from plant cell walls, and can synthesize phytohormones to stimulate plant growth (53). Interestingly, a recent study associated abundances of *Flavobacterium* in wheat rhizospheres with fungal pathogen infection despite noting one outlier *Flavobacterium* OTU associated with healthy plants (54). While saprophytic members of *Cellvibrio* are capable of degrading diverse plant cell wall polysaccharides, at least one member can fix nitrogen (55, 56). Intriguingly, genera with multiple growth-negative ASVs identified in this study (*Adhaeribacter* and *Pedobacter*) have been associated with growth promotion in other rhizosphere systems (54, 57, 58). Within and across studies, the variable influences of rhizobacterial genera on plant health and growth appear to encompass a diversity of context-dependent host-bacterium interactions at finer taxonomic scales.

While treatment dramatically shaped co-occurrence patterns in rhizosphere communities, we detected no differences in connectivity between growth-positive or growth-negative ASVs, in contrast to Jin et al. (31). Different patterns of co-occurrences between growth- and non-growth-associated ASVs across treatments (Fig. 6C) suggests that higher connectivity is associated with growth promotion at the community level, although growth-positive and growth-negative ASVs from both treatments that did not show strong co-occurrences appear to be exceptions to this rule. We suggest that growth-positive ASVs with few strong co-occurrences represent promising candidates for isolation and inoculation to improve plant yields. In contrast, bioinoculation with features showing strong co-occurrences may require characterizing their mechanisms of interaction, designing synthetic communities, and/or directly inoculating seeds with growth-promoting soils.

In summary, we present differing effects of stochastic and deterministic rhizobacterial community assembly processes during a time series of *B. rapa* inoculated with a live or a knocked down soil treatment, noting spatial and temporal heterogeneity. By using a regularized regression approach, we identify correlations between relative abundances of ASVs or modules and several measures of plant growth in early or late stages of *B. rapa* development. Although the associations we report are correlative, different tests identified many of the same features. These associations provide hypotheses for laboratory and field studies on whether and how these rhizobacteria promote plant growth and associate with other members of rhizosphere communities. Because many rhizobacteria remain uncultured or resistant to cultivation, culture-independent approaches such as genome-resolved metagenomics and metatranscriptomics can be used to identify adaptation strategies of recalcitrant populations in plant-associated systems (59). Further investigations should include comparative genomic investigations to characterize variability in growth association patterns among members of influential rhizosphere genera and link changes in rhizosphere communities with plant gene expression and root exudation patterns. A more complete understanding of spatial, temporal, and taxonomic heterogeneity in the rhizosphere will provide a valuable bioengineering foundation for a diversity of agricultural contexts.

## MATERIALS AND METHODS

### Greenhouse experiments.

To explore the timing of rhizosphere formation and its influence on plant growth, we initiated a time course greenhouse experiment at the University of Wyoming in December of 2018 (Laramie, WY; GPS location, 41.319787, −105.558253; elevation, 2,149 m). We planted 204 replicate pots of each of two treatments (disrupted control versus inoculated) with the *Brassica rapa* self-compatible Wisconsin FastPlant (FPsc; Rick Amasino and Scott Woody, University of Wisconsin). Pots in both treatments were filled with a soil matrix consisting of 50% (vol/vol) fritted clay (Profile porous ceramics greens grade; Profile, Buffalo Grove, IL, USA) to 50% sphagnum potting mix (Redi-Earth; Sungro Horticulture, Agawam, MA, USA) amended with powdered organic alfalfa meal (3.5 mL/liter) and adjusted to a pH of 7.0 with powdered lime. The soil matrix was sterilized with two 1-h autoclave cycles separated by 24 h, which has been shown to prevent amplification of extracted DNA (26). For the control (disrupted) treatment, autoclaved bags of soil matrix were opened to atmospheric inoculation in the greenhouse 72 h prior to planting seeds, allowing for colonization by the ambient microbial community. The day before planting, the inoculated treatment was initiated by mixing 2 mm sieved soil from a Wyoming site (South Brush Creek, or SBC; GPS location, 41.327404, −106.502784; elevation, 2,570 m) with sterile autoclaved soil matrix (5%, vol/vol, SBC to matrix). This site is a disturbed montane forest plot where *Boechera stricta* (*Brassicaeae*) is common. Inoculation experiments with *Boechera stricta* and *Brassica rapa* have previously demonstrated significant plant growth promotion using this microbial community (C. J. Hubbard, R. McMinn, M. T. Brock, and C. Weinig, unpublished data).

Pots (8.9 by 8.9 cm, 500 mL) were filled with either control or inoculated soil. The following day, FPsc seeds were sterilized by vortexing for 1 min in 70% ethanol (EtOH), 10 min in 10% bleach, and 5 rinses in sterile water, followed by planting in a 4-by-4 grid (for pots randomly assigned to early sampling days) or a 3-by-3 grid (for pots randomly assigned to late sampling days) and topped with autoclaved vermiculite. Pots with a maximum of 16 seedlings were necessary to attain sufficient rhizosphere mass at early growth stages. Pots were assigned unique locations in alternating cells of 3-by-6 trays, which were arranged into 6 spatial blocks. Greenhouse ambient temperatures were set to 20°C and 15.5°C (day/night), and supplemental LED lights were set to 14-h days (starting at 0500). Germination was first noticed 4 days after planting, which we used as time zero (*T*0; 55% and 33% of seeds germinated 4 and 5 days after planting, respectively).

Six pots of each treatment (1 randomly selected replicate per treatment from each of six spatial blocks) were sampled in the afternoon (1400 h) of days 2, 3, 4, 6, 8, 10, 13, and 14 (Fig. 1). Nested within this 2-week sampling period, we also performed 48-h diel sampling events of 6 pots per treatment at early (days 3 and 4) and late stages (days 13 and 14) at the following additional time points over both days: 0900, 1800, 2200, and 0400. Seedlings within a pot were split between harvesting the rhizosphere (described below) or harvesting root tissue for transcriptome sequencing (RNA-seq) (not presented here). Pots destined for sampling later in the experiment were thinned down after 7 days to a final density of two plants per pot.

To harvest rhizospheres, we gently divided pots in half and pulled intact seedlings and roots from the bulk soil. Roots and rhizospheres were gently shaken to remove residual bulk soil, and then larger soil clumps of >2 mm were removed by hand. Gloves were changed and sterilized with 70% EtOH between handling pots. Roots plus rhizospheres were then placed in microcentrifuge tubes containing DNA/RNA Shield (Zymo Research Corporation, Orange, CA, USA) and placed on a vortexer for gentle disruption of the rhizosphere and mixing with DNA/RNA Shield. Samples were placed in a −80°C freezer until 16S amplicon preparation.

### Plant growth measurements.

To estimate average plant leaf area throughout the experiment, trays of pots were photographed (almost daily throughout the time series) using a dSLR camera at a fixed height on a copy stand. Resulting images were corrected for lens distortion in Photoshop CS6 (Adobe, San Jose, CA, USA) and then cropped to individual pots using custom Perl scripts and ImageMagick (60). Images of individual pots were batch processed with Easy Leaf Area (61) to extract the area of green pixels as our proxy for plant size after dividing green pixels per pot by the number of seedlings present. The relationship between this calculated projected area (PA) and the true leaf area should diminish over time, as plant architecture and leaf self-shading cannot be corrected from a single photo; however, correlations between average PA and average biomass remained highly significant on day 14 (*r* = 0.67; *P* < 0.0001). In addition to PA, we retained aboveground tissue when rhizospheres were harvested throughout the experiment. Tissue samples from each pot were dried for 48 h and weighed, and seedling number was tallied, allowing us to determine average aboveground biomass. Biomass and PA values were reported as means from all plants per pot. We calculated residuals for aboveground biomass and PA to control for differences between days 3 and 4 or 13 and 14 among treatments. Relative growth rates (RGR) were calculated by log transforming PA measurements and calculating slopes from consecutive intervals on a per-pot basis.

### DNA extraction and sequencing.

Genomic DNA templates were prepared using the ZymoBIOMICS DNA/RNA Miniprep kit protocol according to the manufacturer’s instructions (Zymo Research Corporation) using a parallel purification to extract RNA. We followed 16S rRNA gene amplification methods as previously described (62, 63). The starting master mix for the PCR contained 1× SuperFi buffer, 200 μM deoxynucleoside triphosphates, 2.5 U of SuperFi polymerase, and molecular biology-grade water. Before adding template DNA, we added individually indexed fusion primers (0.3 μM) to amplify the bacterial V4-V5 region of the 16S gene (Escherichia coli nucleotides [nt] 518 to 926, 408 nt). Primer sequences are described in reference 62. This volume was mixed by pipetting and 25 μL removed to serve as the negative control. One to 10 ng of template DNA was added to the remaining volume and mixed in by pipetting. The 100 μL was divided into triplicate reaction mixtures. Amplification conditions were an initial denaturation at 94°C for 3 min; 30 cycles of 94°C for 30 s, 57°C for 45 s, and 72°C for 1 min; and a final extension at 72.0°C for 2 min. Triplicate PCRs were pooled and the products cleaned using 0.75 volume of AMPure XP beads (Beckman Coulter) to 1 volume of sample.

Amplicon concentrations were quantified using the PicoGreen assay (ThermoFisher Scientific) and pooled in equimolar amounts based on PicoGreen results. Pools were size selected using BluePippin (Sage Scientific) to a range of 425 to 625 bp on a 1.5% gel cassette. The size-selected products were cleaned again using AMPure XP at a 1:1 ratio and the final pools visualized on an Agilent Bioanalyzer using the DNA1000 assay. The pool concentration was calculated using quantitative PCR (KK4835; KAPA library quantification kit) and the libraries sequenced on an Illumina MiSeq using the version 3 protocol to generate paired-end 300-nt reads.

### Sequence processing and diversity analyses.

DADA2 (64) was used to filter raw 16S fastq reads, infer ASVs, and remove chimeric sequences in a workflow similar to that of Callahan et al. (65). Taxonomy was assigned using version 138 of the SILVA nonredundant (SSU Ref NR99) 16S database (66). ASVs assigned to Eukaryota, mitochondria, and chloroplasts, as well as those that could not be classified at the domain level, were removed using the subset_taxa function in phyloseq (67). Before removal, mitochondria amounted to 2.5% of reads and chloroplasts to 0.7%. Next, contaminant ASVs were removed using the “combined” method of the R package decontam (68), which identified 77 sequence variants that were more abundant across three PCR blanks and in prepooled libraries with lower amplicon concentrations. After removal, the three PCR blanks (2,653 to 4,828 reads each) were pruned using phyloseq, leaving 20,961 ASVs in 293 communities, each sequenced at a depth of 25,438 reads. Alpha diversity calculations were performed using built-in phyloseq functions. Bray-Curtis dissimilarities calculated from cumulative sum scaling transformations of raw read counts (69) were used for PERMANOVA tests and ordinations implemented with the vegan R package (70). A maximum-likelihood phylogenetic tree was constructed in phangorn (71) using the Jukes-Cantor model and 100 bootstrap replications.

### Differential abundance analysis.

A first-order attempt to identify ASVs associated with plant growth promotion yielded no differentially abundant sequences across rhizosphere communities from inoculated-treatment plants based categorically on whether they were much larger, slightly larger, or no larger than their disrupted-treatment counterparts across the entire time series. DESeq2 (33) was used to identify differentially abundant ASVs across soil inoculation treatments and blocks using a threshold *P* value of 0.05 adjusted for multiple comparisons. To search for differentially abundant ASVs across inoculated plants (categorized as much larger, slightly larger, or no larger than their disrupted-treatment counterparts), we calculated aboveground biomass and PA at harvest values of inoculated plants as a percentage of their corresponding disrupted-treatment time point. This yielded three categorical groups: promoted (greater than 107%, the mean normalized score of the inoculated group, *n* = 23), possibly promoted (100 to 107%, *n* = 101), and not promoted (below 100%, *n* = 19). No differentially abundant ASVs were detected across these three groups (comparing communities from each individual group to those in the other two, using DESeq2 with an adjusted *P* value cutoff of 0.05).

### Network analysis.

We constructed cooccurrence networks of the most abundant and prevalent ASVs for six sample groups using SPIEC-EASI (72). These groups, split by treatment, consisted of all samples across the time series or those from days 3 and 4 (early) or 13 and 14 (late). ASVs were included for network analysis if they were detected in at least four communities and their mean abundance was greater than 0.5% within their corresponding sample group (e.g., early disrupted). This resulted in networks that contained 423 to 787 ASVs (Table 1). Associations between covarying ASVs were inferred with SPIEC-EASI using the “glasso” probabilistic inference method and a lambda.min.ratio of 0.01, and significant covariances were converted to correlations with the function cov2cor in a method similar to that of Lemonnier et al. (73). To define modules of highly correlated ASVs, negative edges of networks were removed and the Louvain algorithm (74) was implemented using the cluster_louvain function in igraph (75). igraph was also used to plot and subset networks (without negative edges removed) within customized R functions. Subsetted networks shown in Fig. 6 contain edges with weights below the first quartile value (strongly negative co-occurrences) or edges with weights above the third quartile (strongly positive co-occurrences). First and third quartile values were −0.073 and 0.152 for the inoculated network and 0 and 0.119 for the disrupted network, respectively.

### Feature selection.

We used regularized regression to identify associations between ASV abundances in rhizosphere communities and corresponding plant growth data. This consisted of a generalized linear model (GLM) with a penalized maximum likelihood paired with a least absolute shrinkage and selection operator (LASSO) used to perform variable selection and minimize spurious correlations and overfitting (76). Due to the sparsity in read count data and the low statistical power associated with low-prevalence features, we restricted the GLM input to include only the 200 most abundant ASVs in each sample group that were detected in at least 30% or 50% of communities for disrupted or inoculated treatments, respectively. These prevalence cutoffs were determined heuristically after inspecting rank versus prevalence and cumulative abundance plots of most abundant ASVs across all sample groups (see Fig. S4 at https://doi.org/10.6084/m9.figshare.c.5787851.v1). While imposing different prevalence cutoffs to vary the number of input features, we noticed that a minority of runs returned different numbers of features at different prevalence thresholds. For this reason, we additionally imposed stricter prevalence cutoffs of 50% or 90% for disrupted or inoculated sample groups, respectively, which reduced the number of input features from 200 to 120 to 130 (ASVs with prevalence below 70% were almost never identified, regardless of treatment). The top 200 ASVs in each sample group comprised 78% to 90% of the reads in these communities. Modules of cooccurring ASVs were run with the same growth data as the ASV data without subsetting by prevalence or abundance, because each sample group consisted of fewer than 200 modules. (In the one exception, early inoculation, we omitted 22 modules that were each present in less than 27% of samples and totaled an average of 0.2% of the reads in these communities).

The GLM was implemented with the cv.glmnet function of the R package glmnet (76) using a Gaussian distribution family within a customized R function. The customized R function was used to input raw read counts and corresponding plant growth data from samples (from the otu_table and sample_data of a phyloseq object) as well as parameters to include features based on rank and prevalence and in specifying the number of lasso runs. Read counts were transformed using a centered log ratio (77), and reads of low-prevalence or low-abundance ASVs were summed together to preserve data compositionality.

We found slight variation among the ASVs identified with the GLM LASSO. We suspect that the highly correlated ASV abundances make each run sensitive to initial model conditions. To account for the variation among model runs, we ran each GLM LASSO model 100 times. Features with nonzero coefficients in at least 80 of 100 runs were considered “selected” and further evaluated in our Bayesian hierarchical models (described below). For both ASVs and modules, an optimal number of features corresponding to lambda values that minimized mean squared error during cross-validation was found to be 7 or 8 (for PA and RGR measurements) or 2 or 3 (for PA or biomass residuals).

Using the brms R package (78), Bayesian models of plant growth were constructed using abundances of individual features or combinations of features identified by the GLM. Our rationale for using an additional Bayesian model after the GLM LASSO was due to the constraints and benefits of glmnet: the glmnet function allowed us to efficiently evaluate different ASVs on plant growth but does not allow us to include the block structure in our growth experiments. Bayesian models allowed us to incorporate the block structure of our growth experiments using a hierarchical linear model with a random intercept and slope for each block the plants grew in. A Gaussian family distribution was used to fit response (plant growth) data, and features whose regression coefficient posterior distributions (95% credible intervals) did not include zero were considered selected. Bayesian multivariate models were compared and refined using a leave-one-out cross-validation approach with the loo R package (79). In the ASV data set, 89 ASV growth associations from 73 unique ASVs were reduced to 59 after univariate Bayesian modeling, yielding 30 positive associations with growth and 29 negative. Likewise, the number of module-growth associations was winnowed from 12 to 10 after these respective steps, resulting in six positive and four negative associations. Twelve ASVs and one module were identified as significant by multivariate Bayesian modeling but not individually; on the contrary, 23 ASVs and one module were detected with univariate Bayesian modeling only (Fig. 3 and 5). All features and corresponding sample groups and growth metrics identified across different steps are included in Table S2 (https://doi.org/10.6084/m9.figshare.c.5787851.v1). Univariate Bayesian models preferentially upheld associations between ASVs and growth data identified by the GLM using both prevalence thresholds (14 of 22, 63.6%) compared to those found at only one prevalence (25 of 68, 36.7%). Multivariate Bayesian models explained anywhere from 9% to 80% of the variance in growth (marginal *R*^2^ values), varying with the number of features used in the model. Several models accounting for relative growth rates explained less variance than other growth measurements. The percentages of variance explained by different models did not vary depending on whether a feature was a module or an ASV or by sample group. Model summary statistics are included in Table S3 (https://doi.org/10.6084/m9.figshare.c.5787851.v1).

To examine the specificity of the GLM at identifying spurious features, we randomly scrambled associations between plant growth data and feature abundances and reran all tests. In doing so, we identified 41 associations compared to the 141 identified by the GLM in the unscrambled data. After following up with univariate Bayesian modeling, this number was reduced to 33 (compared to 69 in the unscrambled data). When scrambled, four tests that identified features in unscrambled data identified more than one feature, corresponding to a false discovery rate of over 1% (Table S4 at https://doi.org/10.6084/m9.figshare.c.5787851.v1). Thus, we conservatively decided to remove all associations from these tests that were detected in the unscrambled data.

### Data availability.

Supplemental figures and tables are publicly available as a FigShare collection at https://doi.org/10.6084/m9.figshare.c.5787851.v1. Reproducible code for all analyses, including customized R functions and plant growth data files, are freely available as an archived GitHub repository at https://loimai.github.io/rhizosphere_rapa_16S. Raw fastq sequences of 16S amplicons were deposited to the NCBI sequence read archive (SRA) database under BioProject accession number PRJNA765038.
